# Two-Stage Multi-Task Representation Learning for Synthetic Aperture Radar (SAR) Target Images Classification

**DOI:** 10.3390/s17112506

**Published:** 2017-11-01

**Authors:** Xinzheng Zhang, Yijian Wang, Zhiying Tan, Dong Li, Shujun Liu, Tao Wang, Yongming Li

**Affiliations:** 1 College of Communication Engineering, Chongqing University, Chongqing 400044, China; 20161202029t@cqu.edu.cn (Y.W.); 20161202031t@cqu.edu.cn (Z.T.); ly007@cqu.edu.cn (S.L.); yongmingli@cqu.edu.cn (Y.L.); 2 Key Laboratory of Aerocraft Tracking Telementering & Command and Communication, Chongqing University, Chongqing 400044, China; dongli1983@cqu.edu.cn (D.L.); wt1977@cqu.edu.cn (T.W.)

**Keywords:** SAR, images classification, multitask learning, sparse representation, collaborative representation

## Abstract

In this paper, we propose a two-stage multi-task learning representation method for the classification of synthetic aperture radar (SAR) target images. The first stage of the proposed approach uses multi-features joint sparse representation learning, modeled as a $\ell_{2,1}$-norm regularized multi-task sparse learning problem, to find an effective subset of training samples. Then, a new dictionary is constructed based on the training subset. The second stage of the method is to perform target images classification based on the new dictionary, utilizing multi-task collaborative representation. The proposed algorithm not only exploits the discrimination ability of multiple features but also greatly reduces the interference of atoms that are irrelevant to the test sample, thus effectively improving classification performance. Conducted with the Moving and Stationary Target Acquisition and Recognition (MSTAR) public SAR database, experimental results show that the proposed approach is effective and superior to many state-of-the-art methods.

## 1. Introduction

Synthetic aperture radar (SAR) are widely applied in various civil and military fields, such as aerial remote sensing to detect targets [1], environmental monitoring [2], and maritime surveillance [3]. Automatic target-recognition systems using SAR sensors continue to be developed for a number of applications, particularly in the area of military defense. The goal of these systems is to detect and classify military targets using various image- and signal-processing techniques. The conventional architecture of target-recognition systems consists of three separate stages: the pre-screener identifies local regions of interest using a constant false alarm rate (CFAR) detector, allowing all targets and numerous false alarms to pass. It is followed by a discriminator that aims to eliminate all natural false alarms. Finally, the classifier receives all man-made objects and attempts to categorize each input image as a specific target type contained in the training set [4]. General reviews of automatic target-recognition concepts and the SAR target-detection technologies can be found in [4,5]. This paper focuses on the final classification stage of the SAR automatic target-classification system. Target images obtained by SAR are significantly different with target optical images because microwave imaging is based on a scattering mechanism. Usually, SAR images are not as intuitive and fine as optical images. Moreover, the characteristics of SAR target images are very sensitive to azimuth and elevation angles [4]. If these angles change significantly, the characteristics of the SAR target images also change significantly, which is challenging for their classification.

There are three mainstream paradigms governing SAR target images classification: template-based methods [5], model-based methods [6], and machine–learning based methods [7]. The template-based methods compare the test sample image with training sample images to determine the target category. These methods require a large number of target image templates and expensive computation. Moreover, background clutter also creates greater interference in template matching. For the model-based method, target images are described using a complex scattering model based on a scattering mechanism, and the target type is determined according to the likelihood estimation of the model parameters. However, since these complex scattering models are non-linear, it is difficult to obtain accurate estimation of the parameters. This method is easily affected by clutter interference and noise corruption. In addition, performance of the method at extended operating conditions will significantly decrease because of the obvious difference between training samples and test samples.

Recently, machine-learning based methods have attracted apparent interest for their use in SAR target images classification. One of the most popular methods is representation learning [8,9]. The most typical representation learning method is sparse representation. The sparse representation of one signal is based on the following theory: the observed signal can be represented by a linear combination of a series of known signals called atoms. Under sparse constraints ($\ell_{1}$-norm minimum) of the representative coefficient vector, a unique representative coefficient solution can be obtained, and the target type can be identified according to the minimum reconstruction error for each class. Sparse representation has been widely applied in a variety of recognition tasks, such as face recognition [10,11], speech recognition [12], and hyperspectral image classification [13]. For SAR target images classification, some researchers have investigated a number of approaches based on sparse representation. Zhang et al. proposed a method of multi-view joint sparse representation by exploring the correlation between target multi-view images [14]. Combining low-rank matrix recovery, Cheng et al. proposed an improved joint sparse representation approach to classify SAR target images [15]. Dong et al. investigated a method based on joint sparse representation with monogenic features, and developed the method on a Grassman manifold [16,17]. Furthermore, Liu et al. studied the Dempster–Shafer fusion of multiple sparse representations for target images recognition [18]. Sun et al. proposed a SAR target-recognition method combined with dynamic sparse representation and dictionary learning [19]. Song et al. reported an approach of supervised discriminant dictionary learning and sparse representation for SAR target recognition [20]. Lately, Liu et al. developed a new scatter center feature-extraction and target-recognition method based on sparse representation and a refinement dictionary [21].

Another alternative method of representation learning is collaborative representation [22]. Collaborative representation can be interpreted as an optimization problem under $\ell_{2}$-norm minimum constraints. It has also been widely used in face recognition, hyperspectral classification, etc. [23]. Compared to sparse representation, collaborative representation can greatly reduce the computational cost while maintaining high classification accuracy [23].

In recent years, the combination of multi-task learning and representation learning has become an important trend in pattern recognition. The idea behind multi-task learning is that when these tasks are similar enough or correlative to a certain extent, exploiting the correlation between them is beneficial for improving the generalization of recognition. Some research results have confirmed the advantages of multi-task learning [24]. Yan et al. extracted various types of features from images, and viewed each type’s sparse representation recognition as a task in order to model multi-feature joint sparse representation recognition as a multi-task sparse learning problem [25]. Fang et al. proposed a face-recognition method based on local Gabor feature adaptive multi-task sparse representation [26]. Li et al. proposed a hyperspectral image-classification method by combining multi-task learning with collaborative representation [27]. Luo et al. investigated a manifold regularized multi-task learning (MRMTL) algorithm, which can effectively control the model complexity and ensure that the functions in the shared hypothesis space are smooth along the data manifold [28]. Furthermore, Luo et al. proposed a novel large margin multi-modal multi-task feature extraction framework, which can not only handle correlated and noisy features but also utilize the complementarity of different modalities to reduce feature redundancy [29]. Both multi-task sparse representation and multi-task collaborative representation can exploit shared training sample patterns between different tasks to ensure that the correct training samples are selected, excluding interferential atoms. In addition, recently some SAR target-recognition technologies have been based on deep learning. Ding et al. developed the target-recognition algorithm combined with a data enhancement convolutional neural network [30]. Chen et al. investigated the application of deep convolution networks for SAR target recognition in detail [31].

**Figure 1:**
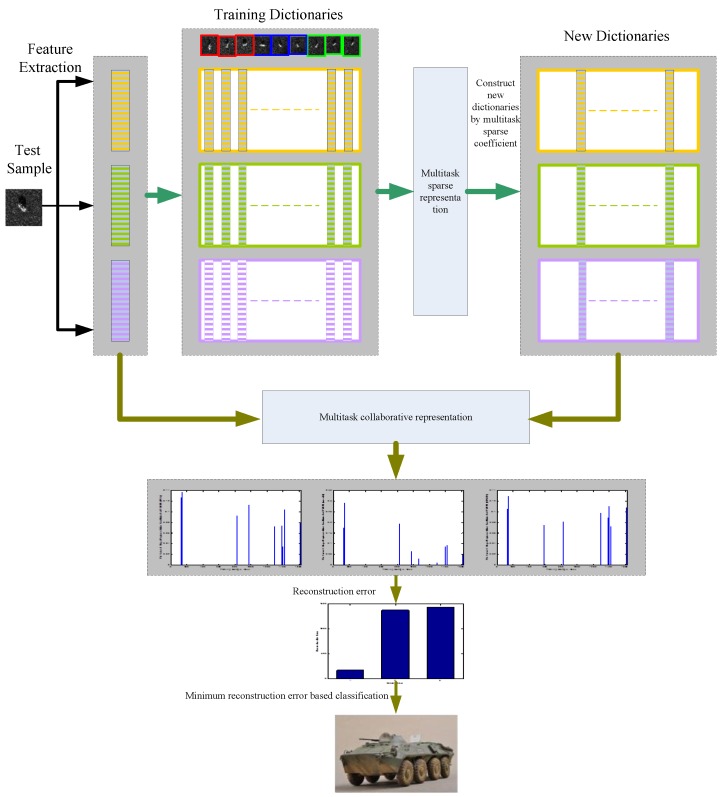
Flowchart of two-stage multi-task representation learning for synthetic aperture radar (SAR) target images classification.

In this work, we propose a two stage multi-task representation learning method for SAR target images classification. Figure 1 shows a schematic view of the approach. The approach first extracts three kinds of features for all training samples and test samples, which include principal component analysis (PCA) features, wavelet transform features, and 2D Slice Zernike Moments (2DSZM) features [32]. The first stage is to represent each feature of the test sample as a linear combination of the corresponding features of the training set, and to determine the Q neighbor samples of the test sample in the training set by using multi-task sparse representation. This is because, in principle, the current test sample and its neighboring samples should come from the same class, which means that Q neighboring samples make the greatest contribution to identifying the test sample. Thus, the first step in this algorithm is to detect training samples that are far from the test sample, it being assumed that these samples have no effect on the classification decision. This is helpful for accurate classification of test samples. In fact, using part of the training samples rather than all training samples to identify a test sample can greatly reduce the interference of those training samples that are far away from the test sample. The second stage of this method is to represent the test sample with the new dictionary consisting of Q neighbors in the framework of multi-task collaborative representation. Furthermore, the representation results are used to infer the test sample label. We adopt multi-task collaborative representation in the second stage because of its uncomplicated closed solution and low computation cost. The proposed method is based on the following factors: the first stage confirms a number of training samples that are most relevant to the current test sample. Since the class labels of the chosen training samples are usually a subset of all class labels, the final classification becomes a problem that determines the test sample label from a small number of candidate class labels. It would be beneficial to have an accurate inference in the second stage that the real label of the test sample was one of the training subset labels. The proposed approach not only exploits the ability of combined multiple features representation learning but also greatly reduces the interference of those irrelevant atoms in the dictionary, which leads to enhanced classification performance. Evaluation of the proposed method is conducted with the Moving and Stationary Target Acquisition and Recognition (MSTAR) benchmark data sets. Experimental results validate the effectiveness and superiority of the proposed approach.

The paper is organized as follows. In Section 2, we briefly describe the three types of feature-extraction methods used in the work, and review basic sparse representation and collaborative representation. In Section 3, a two-stage multi-task representation learning algorithm is developed in detail. In Section 4, experiments are carried out with the MSTAR database, and the performance of the proposed approach is described. Finally, we conclude the paper in Section 5.

## 2. Multiple Features Extraction and Representation Learning Classifier

In this section, we introduce the three types of feature extraction adopted in this paper, and present the sparse representation classifier and collaborative representation classifier.

### 2.1. Multiple Features Extraction

It is well known that each type of feature describes the image from different aspects, and a single feature cannot contain all discriminative information. In addition, it is impractical to extract an optimal feature. For image-classification systems, a better approach is to combine multiple features rather than use a single feature [33]. In this paper, three types of features were extracted from SAR target images, including PCA features, wavelet transform features, and 2DSZM features.

#### 2.1.1. PCA Features Extraction

PCA is a mature feature-extraction technique. The aim of PCA is to find a projection vector, that can represent the original sample in the greatest degree and map the original sample to a low-dimensional space through a linear transformation. Let $y\in R^{1\times N}$ donates the test sample, and $P\in R^{N\times r}$ donates the projection matrix; then, the PCA features extraction can be expressed as follows:

(1) $$s_{\mathrm{PCA}}=yP_{}$$

where $s_{\mathrm{PCA}}$ is the extracted PCA features.

#### 2.1.2. Wavelet Transform Feature Extraction

Wavelet transform is widely applied in image feature extraction, image compression, and so on. In this work, we apply two-dimensional discrete wavelet transform, and vectorize the first-level low-frequency sub-band images as feature vectors. Wavelet feature extraction can be expressed as follows:

(2) $$s_{\mathrm{WAVELET}}=\mathrm{DWT}2(y)$$

where DWT2(•) represents the two-dimensional discrete wavelet transform, and $s_{\mathrm{WAVELET}}$ donates the extracted wavelet transform feature vector.

#### 2.1.3. 2DSZM Feature Extraction

A novel feature named 2DSZM was developed recently, depicting SAR target images scattering features effectively [32]. SAR target images are usually expressed as gray images. According to the electromagnetic scattering theory, each pixel value of a microwave image is related to the intensity of the backscattered wave. In SAR target images, there are strong scattering centers, moderate scattering centers, and weak scattering centers. Since the location and intensity distribution of different scattering regions are not same, different scattering regions can be separated by the slices operation. This operation is called the 2D Slice operation. We extract the Zernike moments of each slice, and then connect all slices’ Zernike moments to get the 2DSZM feature vector donated by $s_{2\mathrm{DSZM}}$. The Zernike moments can be obtained by Equation (3). Details of the 2DSZM feature extraction can be found in the literature [32].

(3) $$Z_{pq}=\frac{p+1}{\pi }\iint_{x^{2}+y^{2}\leq 1}V_{pq}^{*}(x,y)f(x,y)dxdy$$

### 2.2. Sparse Representation Classifier

The success of the sparse signal model can be attributed to the sparse representation of the observed signal with a set of known observation sets (dictionaries). The optimal sparse representation can be obtained effectively by convex optimization methods [34]. We assume that there are k(k=1,…,K) classes of training data, denoted by $X_{k}=\left[x_{k,1},x_{k,2},\ldots ,x_{k,n_{k}}\right]\in$${\mathbb{R}}^{m\times n_{k}}$, where the samples are arranged in ${\mathbb{R}}^{m}$ with the form of column vectors. The unknown observed sample y$\;\in {\mathbb{R}}^{m}$ is represented as a linear combination of the k-th class training samples with the representation coefficient $\alpha_{k}={\left[\alpha_{k,1},\alpha_{k,2},\ldots ,\alpha_{k,n_{k}}\right]}^{T}\in$${\mathbb{R}}^{n_{k}}$.

(4) $$y=x_{k,1}\alpha_{k,1}+x_{k,2}\alpha_{k,2}+\ldots +x_{k,n_{k}}\alpha_{k,n_{k}}$$

Because the class label of the observed sample y is unknown, it is necessary to use all the k classes training samples to represent it. Let $X=\left[X_{1},X_{2},\ldots ,X_{K}\right]\in$${\mathbb{R}}^{m\times n}$, where $n=\sum_{k=1}^{K}n_{k}$ is the total number of training samples. Then, the linear representation of y can be written as follows:

(5) $$y=X_{1}\alpha_{1}+X_{2}\alpha_{2}+\ldots +X_{K}\alpha_{K}=X\alpha$$

where $\alpha ={\left[\alpha_{1},\alpha_{2},\ldots ,\alpha_{K}\right]}^{T}\in$${\mathbb{R}}^{n}$ is the representation coefficient vector. Theoretically, the elements in the vector α corresponding to atoms with the same class as y are non-zero, and other elements are zero. The general method is to find the sparsest representation by adding a regularization term to the representation-coefficient vector [35].

(6) $$\underset{\alpha }{\min}{\left\|\alpha \right\|}_{0}s.t.{\left\|y-X\alpha \right\|}_{2}\leq \varepsilon$$

where ${\left\|\cdot \right\|}_{0}$ represents the $\ell_{0}$-norm of α, and ε donates tolerance error. The optimization of the $\ell_{0}$-norm minimum problem is NP-hard, which is usually relaxed to a $\ell_{1}$-norm minimum problem as follows:

(7) $$\underset{\alpha }{\min}{\left\|y-X\alpha \right\|}_{2}^{2}+\eta {\left\|\alpha \right\|}_{1}$$

where η is the balance parameter, and ${\left\|\cdot \right\|}_{1}$ donates $\ell_{1}$-norm of α. Since Equation (7) is a convex optimization problem, it can be solved by conventional optimization algorithms [36].

After obtaining the optimal sparse representation vector $\hat{\alpha }$, the label of y can be obtained by the minimum reconstruction error discriminant criterion as follows:

(8) $$identity(y)=\underset{k=1,\ldots ,K}{\min}{\left\|y-X_{k}\hat{\alpha }\right\|}_{2}$$

The aforementioned outcome is the famous sparse representation classifier (SRC) [11].

### 2.3. Collaborative Representation Classifier

As an alternative representation-learning theory, the collaborative representation classifier (CRC), has been developed more fully recently [37]. Collaborative representation has been widely used in face recognition, hyperspectral classification, etc. A difference with SRC is that CRC is essentially an $\ell_{2}$-norm optimal problem. The collaborative representation of the observed sample y under the training set X can be written as follows:

(9) $$\overset{⌢}{\beta }=\underset{\beta }{\arg\;\min}\left\{{\left\|y-X\beta \right\|}_{2}^{}+\gamma {\left\|\beta \right\|}_{2}^{2}\right\}$$

where γ is the balance parameter, β is the representation coefficient vector, and ${\left\|\cdot \right\|}_{2}$ donates the $\ell_{2}$-norm of β.

Compared to SRC, the solving process of CRC needs no optimization search; therefore, the computation cost is much lower than SRC. Equation (9) of CRC has a closed solution as follows:

(10) $$\hat{\beta }={\left(X^{T}X+\gamma I\right)}^{-1}X^{T}y.$$

With $\hat{\beta }$, the class label of y can be decided by the minimum reconstruction error criterion as follows:

(11) $$identity(y)=\underset{k=1,\ldots ,K}{\min}{\left\|y-X_{k}\hat{\beta }\right\|}_{2}.$$

## 3. Two-Stage Multi-Task Representation Learning

For image-classification tasks, taking into account the shared patterns among various tasks is beneficial for improving the generalization performance of classification. Numerous studies have confirmed the superior performance of the multi-task learning framework in theory and practice. For example, multi-task sparse representation has been applied in face recognition, and multi-task collaborative representation has been used in hyperspectral image classification. In this work, the classification by each type of feature can be considered as an individual task. Multi-feature classification constitutes a multi-task model, in which multiple tasks should share feature subsets of the training set for a test sample.

Both multi-task sparse representation and multi-task collaborative representation utilize all training samples for classification. However, many studies show that the local subset rather than all of the training samples mainly contributes to classification. That is, many training samples play the role of interference. For instance, it has been illustrated that local PCA is superior to global PCA in classification [38]. Vural proposed the use of local dependencies of samples to conduct the classification [39], while Xu et al. investigated two-phase sparse representation with a local subset of training samples [40].

In this section, we propose a two-stage multi-task representation learning (TSMRL) algorithm for SAR target images classification. The first stage of the algorithm is to represent each feature of the test sample as a linear combination of the corresponding features of all training samples. The $\ell_{2,1}$-norm regularized multi-task sparse learning is adopted to determine the Q nearest neighbor training samples for the test sample. Using local training samples instead of all training samples to identify a test sample can greatly reduce the interference of those training samples far away from the test sample. The new dictionary is constructed by the Q nearest neighbor training samples. In the second stage of the algorithm, multi-task collaborative representation is used for classification with the new dictionary, leading to the final decision of the test sample. In this section, we will describe the proposed two-stage multi-task representation learning method in detail.

### 3.1. The First Stage: $\ell_{2,1}$-Norm Regularized Multi-Task Sparse Representation Learning

Firstly, it is assumed that the test sample features and the training samples features approximately satisfy the following equation:

(12) $$y^{k}=a^{k}{}_{11}x^{k}{}_{11}+a^{k}{}_{12}x^{k}{}_{12}+\ldots +a^{k}{}_{1n_{1}}x^{k}{}_{1n_{1}}+\ldots \ldots +a^{k}{}_{C1}x^{k}{}_{C1}+\ldots +a^{k}{}_{Cn_{C}}x^{k}{}_{Cn_{C}},$$

where $y^{k}\left(k=1,2,3\right)$ here is the *k*-th pattern features of the test sample, and $a_{ci}^{k}(c=1,2,\ldots ,C;i=1,2,\ldots ,n_{c};\;k=1,2,3)$ is the representation coefficient. Equation (12) can be rewritten in the following matrix form:

(13) $$y^{k}=X_{}^{k}a_{}^{k}.$$

According to the sparse representation principle, each pattern feature of the test sample should share features from the same training samples. Taking into account errors caused by noise, we can achieve the following $\ell_{2,1}$-norm regularized multi-task sparse representation model:

(14) $$\arg\underset{A}{\min}\left\{\sum_{k=1}^{3}{\left\|y^{k}-X_{}^{k}a_{}^{k}\right\|}_{F}^{2}+\lambda {\left\|A\right\|}_{2,1}\right\},$$

where λ is the balance parameter, ${\left\|\cdot \right\|}_{F}$ denotes the Frobenius norm of a matrix, and ${\left\|\cdot \right\|}_{2,1}$ denotes the $\ell_{2,1}$-norm, $A=\left[a_{}^{1},a_{}^{2},a_{}^{3}\right]$.

The regularization term in Equation (14) is chosen as the $\ell_{2,1}$-norm regularization term of A. This is because, for all kinds of features from the same test sample, the location of non-zero coefficients in the corresponding sparse coefficient vectors should be similar. Additionally, the corresponding coefficient values of these shared atoms are different due to differences between feature types. Under this assumption, non-zero coefficients of the representation coefficient matrix should be in the same row. A regularized $\ell_{2,1}$-norm can be imposed on A to select a small number of non-zero rows. The optimal problem (14) can be obtainedby the accelerated proximal gradient algorithm [41].

As is known, each column of A is a corresponding representation coefficient vector of each feature. For representing the test sample, each training sample contributes to classification differently. The contribution of a training sample can be estimated by the corresponding representation coefficient value. A large coefficient value means that the training sample makes a large contribution to the representation. Since there are three representation coefficient vectors, we adopt the following method to obtain a local training subset comprising Q nearest neighbor samples. First, the Q atoms with the largest coefficient values are selected in each coefficient vector, leading to three groups. Due to the $\ell_{2,1}$-norm regularized multi-task sparse constraints, most of the Q atoms come from the same training samples. However, there are still a few atoms from different training samples. A new subset will be formed by three groups. We get a subset of V atoms after mixing all three groups and merging the same atoms. We assume that $x_{j}^{k}\left(j=1,\ldots ,V\right)$ is the *k*-th pattern feature vector of the *j*-th atom in the V subset, and let $e_{j}=\sum_{k=1}^{3}{\left\|y^{k}-x_{j}^{k}\right\|}_{2}^{2}$. According to the ascending order of $e_{j}$, we select the first Q atoms from V to form the final nearest neighbor local subset. For each type of feature, the final Q nearest neighbor atoms are reserved, while other atoms are set to zero vectors. In this way, the dictionary $X^{k}$ is updated to a new dictionary $X^{\prime k}$. Thus, the interference caused by irrelevant atoms can be greatly reduced, which can improve correct judgement.

### 3.2. The Second Stage: Multi-Task Collaborative Representation Learning

The second stage of the TSMRL algorithm is to represent the test sample based on the multi-task collaborative representation with the new dictionary $X^{\prime k}$, as follows:

(15) $$\arg\underset{B}{\min}\left\{\sum_{k=1}^{3}{\left\|y^{k}-X_{}^{\prime k}b_{}^{k}\right\|}_{F}^{2}+\rho {\left\|B\right\|}_{F}^{2}\right\},$$

where ρ is the balance parameter. B is the collaborative representation coefficient matrix, and $B=\left[b_{}^{1},b_{}^{2},b_{}^{3}\right]$. The optimal problem of multi-task collaborative representation has analytical solution as follows [27]:

(16) $${\hat{b}}^{k}={\left({\left(X^{\prime k}\right)}^{T}X^{\prime k}+\rho I\right)}^{-1}{\left(X^{\prime k}\right)}^{T}y^{k}.$$

We employ multi-task collaborative representation because of its simplicity in computation and its accurate classification ability.

Then, the class label of y is predicted to the class with the lowest total reconstruction error accumulated over all tasks, i.e.,

(17) $$Class(y)=\underset{c\in \{1,2,\ldots ,C\}}{\arg\min}\sum_{k=1}^{3}{\left\|y^{k}-X_{c}^{\prime k}{\hat{b}}_{c}^{k}\right\|}_{2}^{2}.$$

In summary, the steps of TSMRL are shown in Algorithm 1.

**Algorithm 1 Two-stage multi-task representation learning for SAR target images classification**Input:$X_{m\times n}:$ All training samples$Y_{m\times p}$: All test samplesOutput: the identity of $Y_{m\times p}$ Steps: 1)Extract three types of features from $X_{m\times n}$ respectively; let $X^{1}=X_{\mathrm{PCA}}$, $X^{2}=X_{\mathrm{WAVELET}}$, and $X^{3}=X_{2\mathrm{DSZM}}$.2)Select a test sample $y_{i}$ from $Y_{m\times p}$, and three types of features of $y_{i}$ are extracted; let $y_{i}^{1}=y_{i}{}_{\mathrm{PCA}}$, $y_{i}^{2}=y_{i}{}_{\mathrm{WAVELET}}$, and $y_{i}^{3}=y_{i}{}_{2\mathrm{DSZM}}$.3)Using $\ell_{2,1}$-norm regularized multi-task sparse representation, represent {$y_{i}^{1}$,$y_{i}^{2}$,$y_{i}^{3}$} with the dictionaries {$X^{1}$,$X^{2}$,$X^{3}$}; obtain the representation matrix A. Then, obtain the local subset comprising Q nearest neighbors, and construct new dictionaries {${X^{\prime }}^{1}$,${X^{\prime }}^{2}$,${X^{\prime }}^{3}$}.4)Using multi-task collaborative representation, represent {$y_{i}^{1}$,$y_{i}^{2}$,$y_{i}^{3}$} with the new dictionaries {${X^{\prime }}^{1}$,${X^{\prime }}^{2}$,${X^{\prime }}^{3}$}. Then, obtain the representative matrix B.5)Decide the label of the test sample $y_{i}$ based on the criterion of the total minimum reconstruction error of multi-task collaborative representation.6)If all testing samples are classified, go to step 7). Otherwise, return to step 1).7)End

## 4. Experimental Results

### 4.1. Experimental Data Set

**Figure 2:**
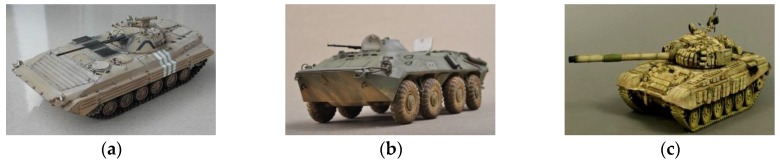
Optical images of three targets. (**a**) BMP2; (**b**) BTR70; (**c**) T72.

**Figure 3:**
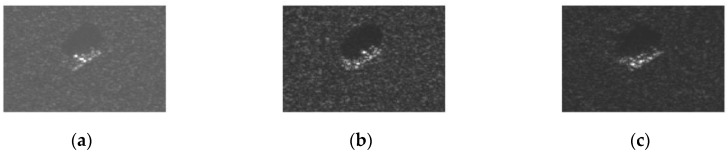
SAR images of three targets. (**a**) BMP2 (45.5°); (**b**) BTR70 (45.0°); (**c**) T72 (44.8°).

In order to evaluate the performance of the proposed approach, we used the MSTAR SAR image database to carry out experiments. SAR is a typical microwave imaging sensor that can obtain high-resolution two-dimensional images of targets. The database is widely used for the performance evaluation of various SAR target-recognition algorithms. The database was acquired by X band SAR sensors with 0.3 m × 0.3 m resolutions. The main targets in the database are armored vehicles, tanks, and other vehicles. SAR images in the database have an azimuth range from 0–360°, and have two different depression angles (15°, 17°). In the experiments, we mainly used images of three targets, namely BMP2, BTR70 and T72. The optical images of the three targets are given in Figure 2. The SAR images of the three targets with similar azimuth angles are given in Figure 3. In particular, BMP2 has three different configurations: BMP2-9563, BMP2-9566, and BMP2-C21. BTR70 has only one configuration, BTR70-C71. T72 also has three different configurations: T72-132, T72-812 and T72-S7. Table 1 lists the number of training samples and test samples. SAR images of the three targets obtained at a 17° depression angle were used as training samples, while images acquired at a 15° depression angle were used as test samples. In addition, a SAR image from the MSTAR database contains background clutter besides the target; and the target is usually located in the central region of the SAR image. In order to reduce the interference of background clutter, we used a 60 × 60 window to get the central region of the original SAR image.

### 4.2. Classification Results and Analysis

We first analyzed the classification performance of the proposed TSMRL algorithm. In order to illustrate the effectiveness of the TSMRL, we compared it to the multi-task sparse representation classifier (MSRC) and the multi-task collaborative representation classifier (MCRC). The advantage of TSMRL lies in the fact that the best local subset for the test sample can be obtained through the $\ell_{2,1}$-norm regularized multi-task joint sparse in the first stage, which can greatly reduce the interference of irrelevant atoms. In fact, this agrees with the characteristics of SAR target images, which are sensitive to azimuth changes. Thus, only training samples from the same target and with adjacent azimuth can be selected as nearest neighbors of the test sample.

#### 4.2.1. Classification Performance under Different Feature Dimensions

**Figure 4:**
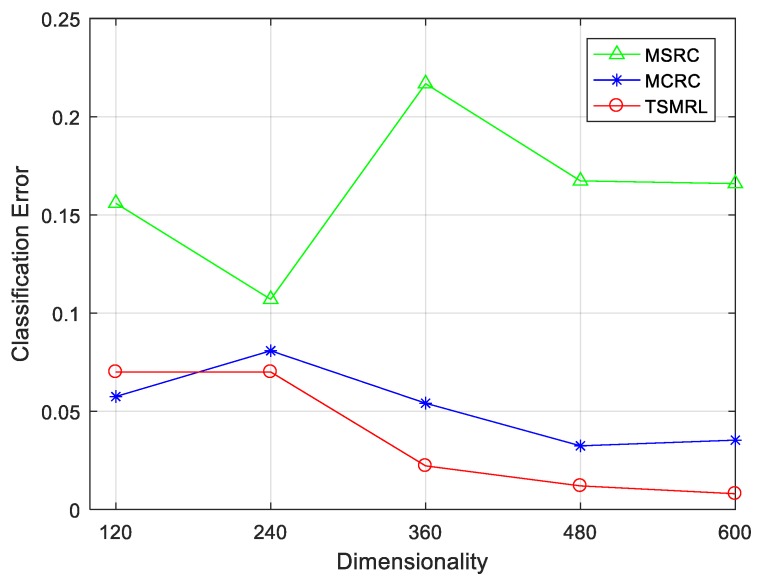
Classification performance of three algorithms with varying feature dimensions.

First, we compared the classification performance of the three methods under different feature dimensions. The results are shown in Figure 4 and Table 2. It is necessary to note that the dimensionality of three features used in this paper is different. Therefore, the change of feature dimensions was obtained by decreasing each feature dimension with the same ratio. The horizontal axis in Figure 4 is the dimensionality of the PCA feature. As can be seen from Figure 4, the error rate of TSMRL decreases with the increase of feature dimension. When the dimension is 600, TSMRL had the lowest error rate of 0.80%, that is, the average classification accuracy was 99.20%. Under the same feature dimension, the error rates of MCRC and MSRC were 3.53% and 16.56%, respectively, which are much higher than that of the TSMRL. Moreover, the TSMRL can maintain small error rates in lower dimensions. These experimental results support the advantages of the proposed algorithm for SAR target images classification.

#### 4.2.2. Classification Performance under Different Number of Neighbor Atoms

**Figure 5:**
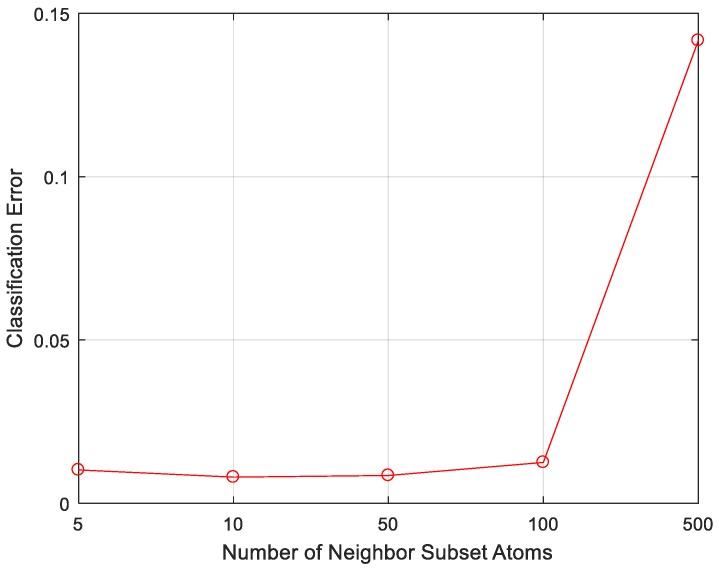
Classification performance of two-stage multi-task representation learning (TSMRL) under different number of neighbor subset atoms.

The dictionaries in both MSRC and MCRC are composed of all training samples. However, in TSMRL, a subset of training samples is selected after the first stage $\ell_{2,1}$-norm regularized multi-task joint sparse learning. Thus, although the dictionary size is the same as those of MSRC and MCRC, the number of valid non-zero atoms is much smaller than those of MSRC and MCRC. We changed the value of Q in order to observe the impact of the number of selected neighbor atoms. The classification results are shown in Figure 5.

As shown in Figure 5, when Q equals 10, the error rate of TSMRL attains the minimum of 0.80%. With the increased Q, the error rate becomes larger, and reaches 14.17% when Q equals 500. This is mainly because many interfering atoms are inevitably introduced with the increased number of neighbor atoms. Nevertheless, it can be seen from Figure 5 that TSMRL still maintains low error rates over a wide range of Q values from 5 to 100. According to the above results, the number of neighbor atoms was set to 10 in subsequent experiments.

**Figure 6:**
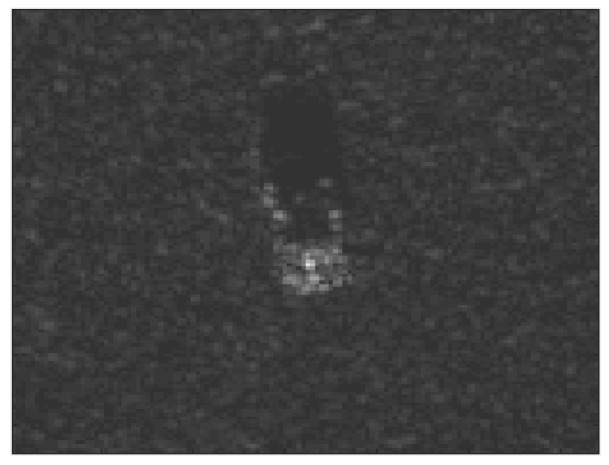
Test sample BMP2-9563 (172.50°).

**Figure 7:**
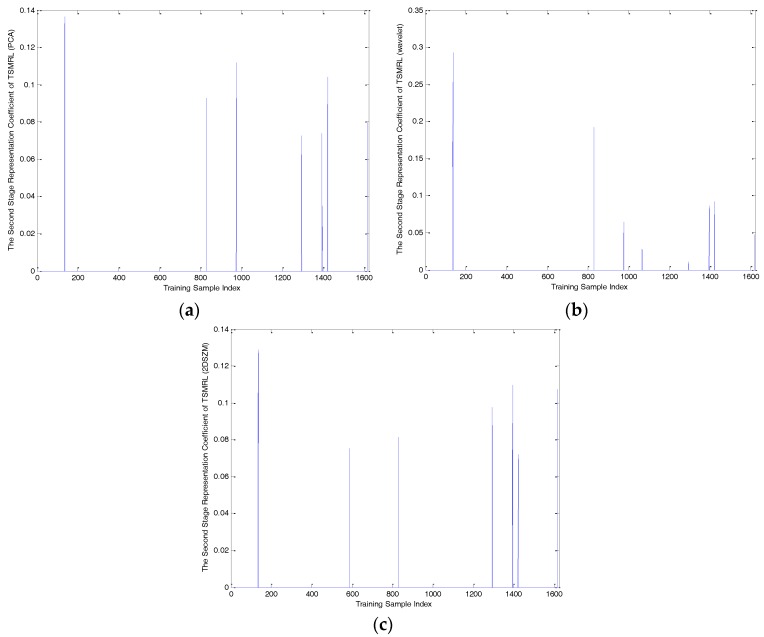
(**a**) The second-stage representation coefficient of principal component analysis (PCA) features of TSMRL; (**b**) the second-stage representation coefficient of wavelet features of TSMRL; (**c**) the second stage representation coefficient of 2DSZM features of TSMRL.

**Figure 8:**
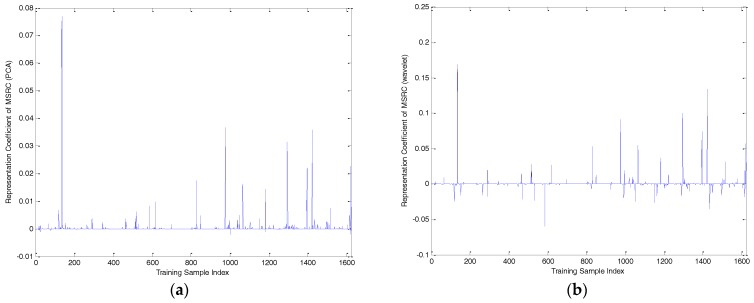

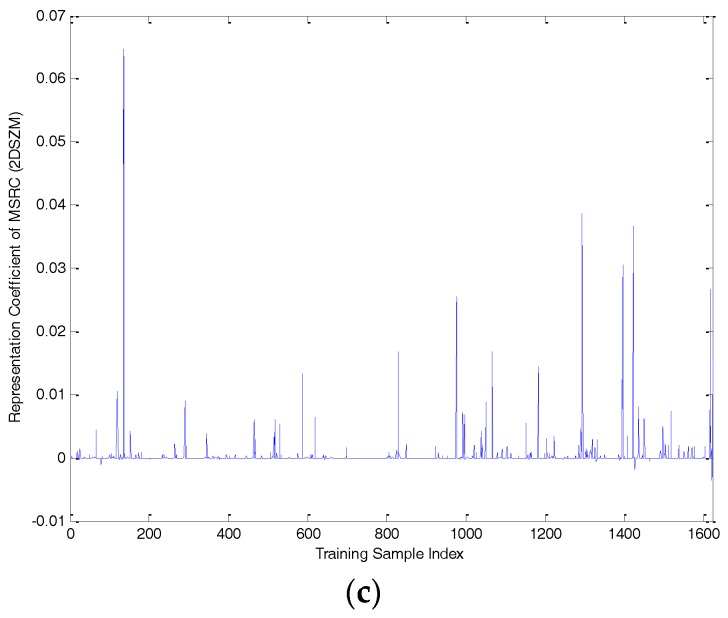
(**a**) Representation coefficient of PCA features of multi-task sparse representation classifier (MSRC); (**b**) representation coefficient of wavelet features of MSRC; (**c**) representation coefficient of 2DSZM features of MSRC.

**Figure 9:**
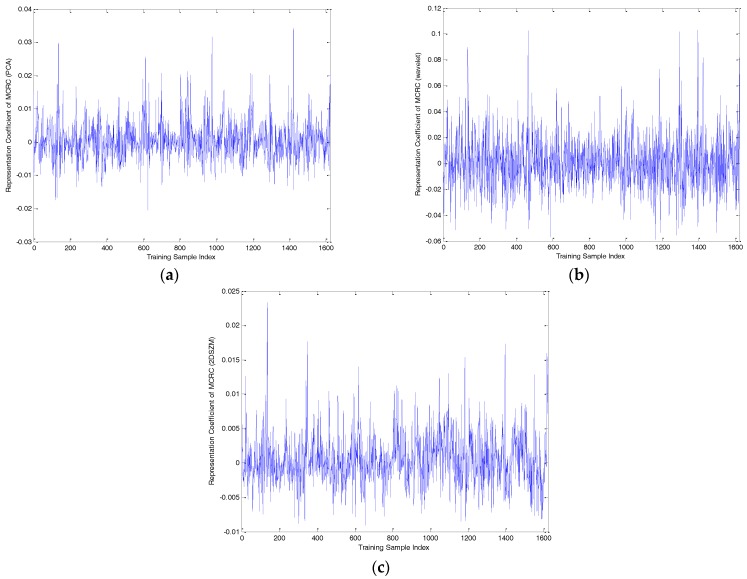
(**a**) Representation coefficient of PCA features of multi-task collaborative representation classifier (MCRC); (**b**) representation coefficient of wavelet features of MCRC; (**c**) representation coefficient of 2DSZM features of MCRC.

**Figure 10:**
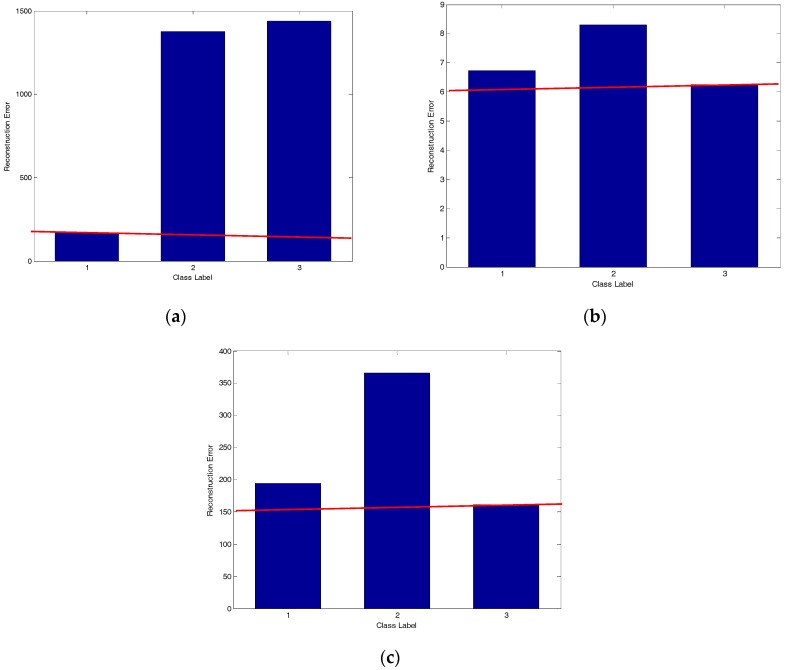
(**a**) Reconstruction error of TSMRL; (**b**) reconstruction error of MSRC; (**c**) reconstruction error of MCRC.

To compare the performance of the three methods definitively, one query sample of BMP2-9563 with an azimuth angle of 172.5° was selected to analyze the representation coefficient and reconstruction error, as shown in Figure 6, Figure 7, Figure 8, Figure 9 and Figure 10. Compared to the representation coefficient of both MSRC and MCRC, the representative coefficient vector of TSMRL becomes much sparser, as shown in Figure 7. The reason for this is that we selected a fixed number of neighbor atoms (Q = 10) for TSMRL. However, for both MSRC and MCRC, there are many atoms with large coefficients belonging to non-BMP2 in the representation coefficient vector, which causes huge interference. The reconstruction error graphs of the three methods shown in Figure 10 confirm our predictions. Figure 10 suggests that minimal reconstruction errors for both MSRC and MCRC correspond to the incorrect class, while the minimal reconstruction error for TSMRL means that it is able to correctly identify the target. As shown in Figure 10a, the reconstruction error of the query sample in TSMRL is far less than other target categories. In summary, TSMRL can identify targets more accurately than MSRC and MCRC.

For MSRC and MCRC, coding was performed across the global azimuth range of 0–360°. For TSMRL, after the first stage $\ell_{2,1}$-norm regularized multi-task joint sparse learning, a subset of training samples was selected. The training samples in the selected subset became the effective atoms, while the other atoms were set to zero vectors. In this way, we constructed a new dictionary, which the second stage was based upon. These selected neighbor atoms play an important role for correct classification. In summary, the experimental results shown in Figure 7, Figure 8, Figure 9 and Figure 10 demonstrate that TSMRL can prevent the interference of atoms far away from the test sample, and improve recognition performance.

#### 4.2.3. Classification Performance with Regularization Parameter Value Change

There are two regularization parameters, λ and ρ, in TSMRL. The values of the two regularization parameters have an important impact on classification performance.

**Figure 11:**
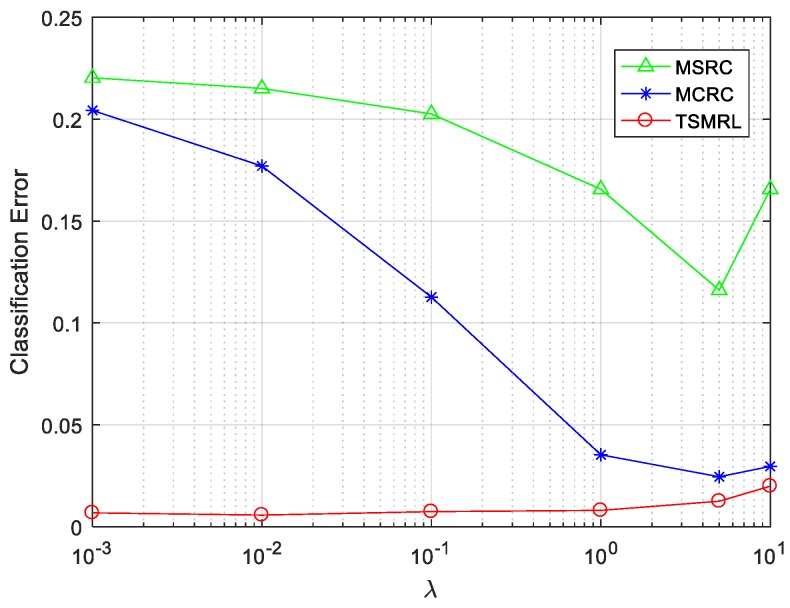
Classification performance of the three algorithms with different values of regularization parameter λ.

There is only one balance parameter in both MSRC and MCRC. In order to compare TSMRL with MSRC and MCRC, two regularization parameters in TSMRL take the same value as those in the other two algorithms. In this experiment, the value of Q was fixed to 10, and the regularization parameter values were set to 10^−3^, 10^−2^, 0.1, 1, 5 and 10 respectively. Figure 11 and Table 3 and Table 4 show the classification error variation diagram, the error rate table, and the confusion matrix, respectively. From the curve of Figure 11, it can be seen that MSRC and MCRC are sensitive to changes of regularization parameter values, and the recognition error rates tend to descend with the increase of the value from 10^−3^ to 5. However, TSMRL is rather smooth with changes of regularization parameter values, and the error rate does not change obviously with the variety of parameter values, which shows that TSMRL had better robustness to various regularization parameters. This is very important in practical applications because you do not always know the ideal regularization parameters. Taking the regularization parameter value 0.1 as an example, the classification error rates corresponding to MSRC, MCRC and TSMRL were 20.26%, 11.27% and 0.74%, respectively. The classification performance of TSMRL was much better than that of MSRC and MCRC. The entire statistical classification error rates are shown in Table 3. It is clear that TSMRL has the highest classification accuracy and small fluctuation. By contrast, the classification performance of MSRC and MCRC is not stable. In addition, it can be seen that when the regularization parameter value is 10^−2^, TSMRL achieves the minimum error rate of 0.57%, i.e., the correct recognition rate is as high as 99.43%. As shown in Table 4, with the regularization parameter value 10^−2^ as an example, the number of correct classified three targets obtained by TSMRL is better than that of MSRC and MCRC under the same conditions. The number of BMP2 correctly identified by TSMRL is 583, which is significantly better than the 453 of MSRC and 560 of MCRC. The results of these experiments illustrate that TSMRL had the best discrimination ability among the three algorithms.

**Figure 12:**
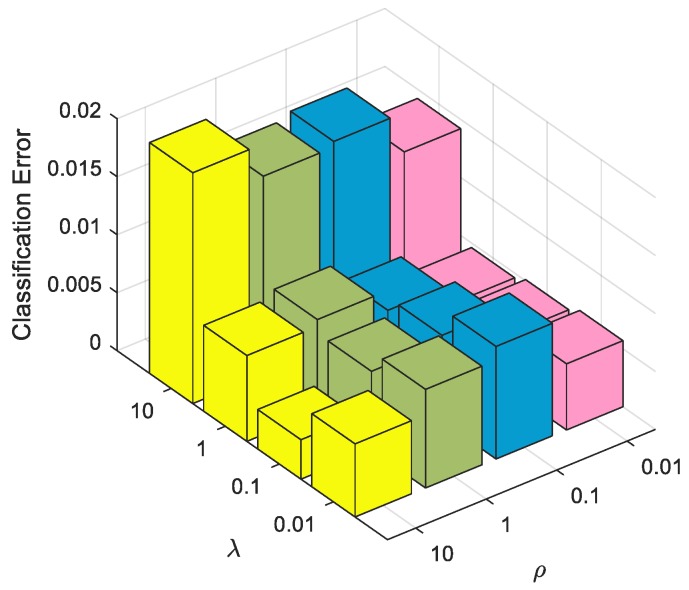
Classification errors of TSMRL with different regularization parameters.

TSMRL uses two-stage multi-task representation learning. The first stage is the $\ell_{2,1}$-norm regularized multi-task sparse learning, the balance parameter of which is λ. The second stage is the Frobenius norm regularized multi-task collaborative learning, and the balance parameter is ρ. In previous experiments, λ and ρ took the same value. However, in practice their values are often inconsistent. Therefore, it is necessary to analyze the classification performance when both values are not equal. We set λ and ρ for different values and carried out the classification experiment using TSMRL. The results are shown in Figure 12. As can be seen from Figure 12, the error rates of TSMRL were lower than 2% when λ and ρ are in the range of 10^−2^–10. The results further illustrate TSMRL’s robustness to a variety of regularization parameter values. On the other hand, when λ is 0.1 and ρ is 10, TSMRL achieves the minimum error rate of 0.34%, i.e., the correct recognition rate was as high as 99.66%. These experimental results support the advantages of the proposed algorithm.

Furthermore, we compared the performance of the proposed method with recent SAR target images classification methods. Under the same dataset, the method in [19] achieved a recognition rate of 96.48%; the approach in [32] achieved a recognition rate of 98.63%. The recognition rate of the proposed method reached 99.66%, which was better than the above methods.

#### 4.2.4. Robustess to Noise

**Figure 13:**
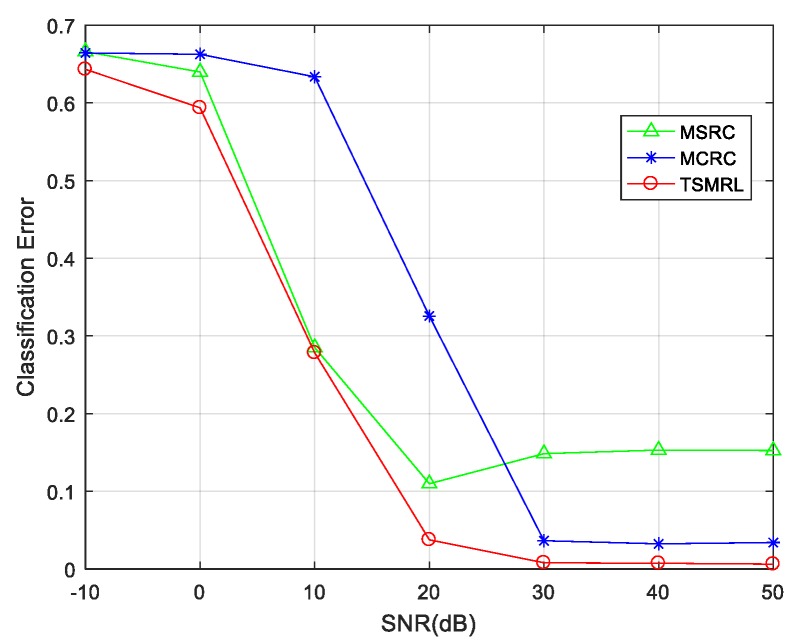
Performance of three algorithms with varying signal-to-noise ratios (SNRs).

Due to the complex imaging environment of the battlefield, it is important to investigate the anti-noise performance of the proposed algorithm. Gaussian noises of different levels with varying signal-to-noise ratios (SNR) were added into testing samples, thereby enabling evaluation of the robustness of the algorithm. Figure 13 shows the classification performance curves of the three algorithms at different SNRs. From the figure, we can see that the error rates of all algorithms decrease with higher SNR. Nevertheless, the TSMRL algorithm has the minimal error rate among all methods for each SNR level. Furthermore, an error rate of 3.67% can be achieved under 20 dB SNR by the TSMRL algorithm, and an error rate of 0.74% can be obtained under 40 dB SNR level, which illustrates its robustness to noise corruption.

#### 4.2.5. Experiments Conducted with Depression Angle Variations

**Figure 14:**
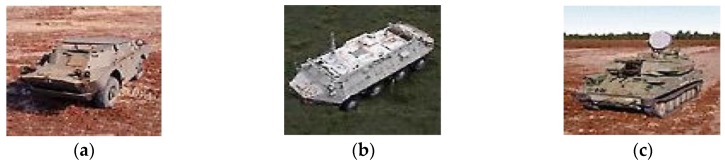
Optical images of three targets. (**a**) BRDM2; (**b**) 2S1; (**c**) ZSU23/4.

**Figure 15:**
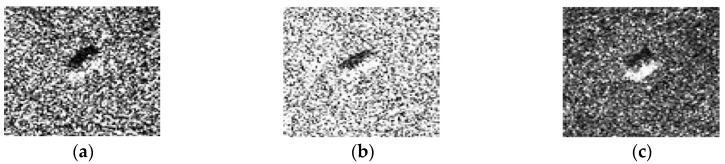
SAR images of three targets. (**a**) BRDM2; (**b**) 2S1; (**c**) ZSU23/4.

In practical applications, SAR images of a target are not collected at a fixed depression angle. When the depression angle of the target changes, the SAR image varies as well. In other words, a change in depression angle also affects the classification performance of the algorithm. Thus, it is necessary to analyze the classification performance with depression angle variations. In our experiments, the three algorithms were evaluated with a large depression angle variation. Three targets, BRDM2, 2S1, and ZSU23/4 were employed, as shown in Figure 14. SAR images of these targets are shown in Figure 15. The dataset used in the experiment is given in Table 5. SAR images collected at a 17° depression angle were used for training, while images collected at 30° and 45° were employed for testing. The experimental results are shown in Table 6.

As shown in Table 6, with a 30° depression angle, the error rate of TSMRL was 5.33%, obviously lower than those of MSRC and MCRC. With the depression angle increasing to 45°, the recognition performance of the three methods all declined. This was mainly because SAR images of the targets changed severely with an angle gap of 28° between training and test samples. However, TSMRL could still achieve the minimum classification errors, which were 19.70% and 15.41% lower than those of MSRC and MCRC, respectively.

The experimental results verify that TSMRL is most robust when it comes to the depression variation among the three algorithms. This is because of the two-stage strategy reducing confusion caused by large depression variation, which ensures accurate multi-task learning.

## 5. Conclusions

In this paper, we proposed a two-stage multi-task representation learning model to classify SAR target images. The proposed method could not only make use of multi-feature collaborative discriminative ability for targets, but could also integrate the representation learning capacity of sparse representation and collaborative representation. The first stage $\ell_{2,1}$-norm regularized multi-task sparse learning selects the nearest subset of the test sample from the perspective of representation learning, which greatly reduces the interference caused by outliers. In this way, a new dictionary is obtained. Through the second stage of multi-task collaborative representation learning, the rational representation weight coefficients are assigned to each atom in the new dictionary. Subsequently, the label of the test sample can be inferred accurately based on the criterion of the minimum reconstruction error. Moreover, because the second-stage multi-task collaborative representation has a closed form solution, the computational complexity of the whole algorithm is mainly embodied in the first stage of multi-task sparse representation learning. The experimental results show that the classification performance of the proposed algorithm is much better than that of multi-task sparse representation learning and multi-task collaborative representation learning, and the algorithm is robust for a wide range of regularization parameters.

## Figures and Tables

**Table 1:** The targets and number of training and test datasets.

Number of Objects	1	2	3	4	5	6	7
Training sample type (17°)	BMP2 sn-9563	BMP2 sn-9566	BMP2 sn-c21	BTR70 sn-c71	T72 sn-132	T72 sn-812	T72 sn-s7
Number	233	232	233	233	232	231	228
Testing sample type (15°)	BMP2 sn-9563	BMP2 sn-9566	BMP2 sn-c21	BTR70 sn-c71	T72 sn-132	T72 sn-812	T72 sn-s7
Number	195	196	196	196	196	195	191

**Table 2:** Error rates of the three algorithms with varying feature dimensions.

Dimensionality	Method		
	TSMRL	MSRC	MCRC
120	7.00%	15.59%	5.75%
240	7.00%	10.70%	8.08%
360	2.22%	21.68%	5.41%
480	1.20%	16.73%	3.24%
600	0.80%	16.56%	3.53%

**Table 3:** Classification error rate of the three algorithms with regularization parameter λ.

λ	Method		
	TSMRL	MSRC	MCRC
10^−3^	0.68%	22.03%	20.43%
10^−2^	0.57%	21.51%	17.70%
0.1	0.74%	20.26%	11.27%
1	0.80%	16.56%	3.53%
5	1.25%	11.61%	2.45%
10	1.99%	16.56%	2.96%

**Table 4:** Confusion matrix of the three algorithms under different regularization parameter values.

**Method**	**Ground Truth**	λ **= 10^−3^**			λ **= 10^−2^**		
		**BMP2**	**BTR70**	**T72**	**BMP2**	**BTR70**	**T72**
TSMRL	BMP2	585	0	2	586	0	1
	BTR70	5	579	4	4	581	3
	T72	1	1	580	1	1	580
MSRC	BMP2	433	91	63	439	86	62
	BTR70	66	487	35	64	495	29
	T72	56	76	450	57	80	445
MCRC	BMP2	548	0	39	552	0	35
	BTR70	168	274	146	145	317	126
	T72	6	0	576	5	0	577
**Method**	**Ground Truth**	λ **= 0.1**			λ **= 1**		
		**BMP2**	**BTR70**	**T72**	**BMP2**	**BTR70**	**T72**
TSMRL	BMP2	583	0	4	583	1	3
	BTR70	6	580	2	8	578	2
	T72	1	0	581	0	0	582
MSRC	BMP2	453	73	61	484	64	39
	BTR70	53	510	25	54	510	24
	T72	65	79	438	55	55	472
MCRC	BMP2	560	0	27	562	2	23
	BTR70	96	421	71	15	552	21
	T72	4	0	578	1	0	581
**Method**	**Ground Truth**	λ **= 5**			λ **= 10**		
		**BMP2**	**BTR70**	**T72**	**BMP2**	**BTR70**	**T72**
TSMRL	BMP2	581	1	5	579	1	7
	BTR70	6	574	8	17	563	16
	T72	1	1	580	2	0	580
MSRC	BMP2	536	32	19	539	9	39
	BTR70	53	505	30	122	381	85
	T72	28	42	512	14	22	546
MCRC	BMP2	554	7	26	542	11	34
	BTR70	0	580	8	0	585	3
	T72	0	2	580	1	3	578

**Table 5:** Dataset used in experiment.

	BRDM2	2S1	ZSU23/4
Training Set (17°)	298	299	299
Testing Set (30°)	287	288	288
Testing Set (45°)	303	303	303

**Table 6:** Classification error under different depression angles of the three methods.

Depression	Method		
	TSMRL	MSRC	MCRC
30°	5.33%	28.85%	8.11%
45°	44.11%	63.81%	59.52%
